# Efficacy of baloxavir marboxil as a prophylactic against influenza among in-patients and analysis of influenza virus mutations in their isolates at a Japanese hospital during the 2023–2024 flu season

**DOI:** 10.1186/s40780-025-00418-5

**Published:** 2025-03-17

**Authors:** Akihide Fujii, Tomohiro Oishi, Eisei Kondo, Zyunko Morihara, Yuka Ikeda, Hideyuki Sumida, Youko Ninomiya, Satoe Uesugi, Kimie Okazaki, Daisuke Yoshioka, Miyoko Kobayashi, Hideho Wada

**Affiliations:** 1 Department of Pharmacy, Kawasaki Medical School Hospital 577, Kurashiki Okayama, Matsushima 701-0192 Japan; 2 Department of Clinical Infectious Diseases, Kawasaki Medical School 577, Kurashiki Okayama, Matsushima 701-0192 Japan; 3 Department of Hematology, Kawasaki Medical School 577, Kurashiki Okayama, Matsushima 701-0192 Japan; 4 Department of Clinical Laboratory, Kawasaki Medical School Hospital 577, Kurashiki Okayama, Matsushima 701-0192 Japan

**Keywords:** Baloxavir marboxil, Hospital, Prophylaxis, Mutation

## Abstract

**Background:**

Baloxavir marboxil (baloxavir) is a new anti-influenza drug that works as a cap-dependent endonuclease inhibitor. It is approved for prophylactic use against influenza in Japan, but there are few reports on this usage in hospitalized in-patients. It reportedly reduces patient susceptibility to influenza through a mechanism involving amino acid substitution.

**Methods:**

Between August 2023 and July 2024, we investigated the efficacy of baloxavir as a prophylactic against influenza among in-patients at our hospital who had close contact with patients who were infected with influenza viruses in the same rooms. We also investigated the I38T influenza virus variant to baloxavir through samples taken from patients with the virus at the hospital.

**Results:**

We enrolled a total of 45 in-patients who had close contact with other patients who were confirmed to be infected with influenza in the same room. Among 34 of them who were prescribed baloxavir prophylactically, none developed influenza within 5 days from their last contact with infected patients. Conversely, among the other 11 who did not use baloxavir, three became infected with influenza within 5 days following contact with the infected patient (*P* = 0.012). In 85 samples taken from the patients with influenza, 25 were H1N1 types and 60 were H3N2. We detected the I38T variant of the cap-dependent endonuclease-baloxavir complex structure in two of the H3N2-type samples. Both of these patients had contracted influenza from their children.

**Conclusions:**

According to our results, Baloxavir represents an effective prophylactic against influenza for hospitalized in-patients. However, patients with genetic mutations related to decreased susceptibility to influenza, such as I38T variant, should nevertheless exercise higher levels of caution—particularly around children.

## Background

Baloxavir marboxil (baloxavir) is an antiviral agent against influenza that works as a cap-dependent endonuclease inhibitor. This differs from most other antiviral agents that target influenza, which function by inhibiting the neuraminidase enzyme.

Baloxavir has been proven to be more effective than oseltamivir, a neuraminidase inhibitor. Specifically, treatment with baloxavir shortened the duration of influenza-related illness by approximately 1 day compared to oseltamivir [1, 2].

Recently, baloxavir was reported to be effective as a prophylactic against influenza in individuals who had close contact with influenza-infected patients within their households [3, 4]. Baloxavir was approved for use as a prophylactic against influenza in 2020 in Japan. However, to the best of our knowledge, there have been no previous reports on its effectiveness for this application in hospitalized in-patients—many of whom are considered at high risk of developing severe influenza. Baloxavir reportedly reduces susceptibility to influenza through a mechanism involving amino acid substitution [5, 6].

In this study, we investigated the efficacy of baloxavir as a prophylactic against influenza in hospitalized in-patients, as well as the frequency of amino acid substitutions involved in its use.

## Materials and methods

### Setting and population

This was a single-center, retrospective study conducted on out-patient and in-patient populations at Kawasaki Medical School hospital, Japan.

We enrolled in-patients who had been in contact with patients who were confirmed to be infected with influenza in the same hospital rooms between August 2023 and July 2024 (i.e., the 2023–2024 influenza season). Throughout this season, all individuals were required to wear masks, and meetings were restricted to two persons in private rooms or in the ward lobby during the afternoon.

Furthermore, we collected samples of influenza from out-patients and in-patients at Kawasaki Medical School hospital.

All of the patients and their family members provided informed consent prior to inclusion in the study, and the study was approved by the Ethics Committee of Kawasaki Medical School (approval no.: 5316-02).

### Data collection

We collected patient data, including age and sex, whether the patient was prescribed baloxavir as an anti-influenza prophylactic, and whether they developed influenza within 5 days following their last contact with an infected person. All of the patients who were prescribed the baloxavir prophylactic received their prescriptions within 24 h after their infected contacts had been diagnosed with influenza.

We also investigated the types of influenza and whether the strains harbored mutations related to baloxavir resistance. All influenza viral samples isolated from the patients at our hospital were investigated for mutations related to baloxavir resistance.

The influenza virus was detected in specimens using nasopharyngeal swabs using a standard influenza virus test via the Lumipulse L2400 instrument (Fujirebio Inc., Tokyo, Japan). For influenza-positive specimens, we further performed virus typing or subtyping analyses using reverse-transcription-quantitative-polymerase-chain-reaction (RT-qPCR) and investigated the presence of the I38T substitution mutation in the polymerase acidic subunit (PA). This was done using RT-qPCR previously reported primers [1] and newly designed locked nucleic acid (LNA)-based TaqMan probes: H3N2_38I (SUN-CA+GCA+A+T+ATGCA+CTC-BHQ), H3N2_I38T (FAM-CAGCA+A+C+ATGCA+CTC-BHQ), H1N1_38I (SUN-CTGCA+A+T+TTGCA+CAC-BHQ), and H1N1_I38T (FAM-CTGCA+A+C+TTGCA+CAC-BHQ).

### Statistical analyses

GraphPad Prism 5 (GraphPad Software Inc., San Diego, CA, USA) was used for all statistical analyses. Differences between pairs of groups were analyzed using Chi-squared, Student’s t-, Fisher’s exact, or Mann-Whitney U tests to determine the 95% confidence intervals. P values of <0.05 were considered to be statistically significant.

## Results

Table 1 shows the details of our cohort of patients with influenza over the 2023–2024 flu season, divided into those who received prophylactic baloxavir and those who did not. The recorded patient data included age, sex, type of influenza at onset, and the presence of secondary influenza.

**Table 1:**
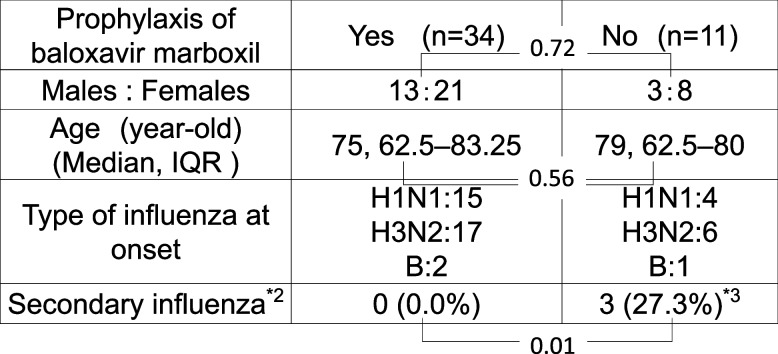
Close contacts of patients with influenza ^*1^, and  secondary influenza infections among them, with and without the prophylactic use of baloxavir marboxil, during the 2023–2024 flue season in Japan

*NS* Not Significant

^*1^Close contacts of patients with influenza were defined as those who had shared a hospital room at the commencement of the study

^*2^Secondary influenza was defined as disease onset among close contacts within 4 days of the last known contact7)

^*3^All of the secondary influenza types were identified as the H3N2 type

Thirty-four patients were prescribed baloxavir prophylactically, whereas 11 were not. There was no significant difference in terms of sex between the patients who received prophylactic baloxavir vs those who did not, but there was a significant difference in the rate of secondary influenza infections between these groups (P=0.012). Specifically, 0.0% (0/34) of the patients who received baloxavir developed secondary influenza, compared to 27.3% (3/11) of those who did not. All of the secondary influenza infections were of the H3N2 type.

The data regarding mutations related to baloxavir resistance among the type A influenza isolates from the patients are presented in Table 2.

**Table 2:**
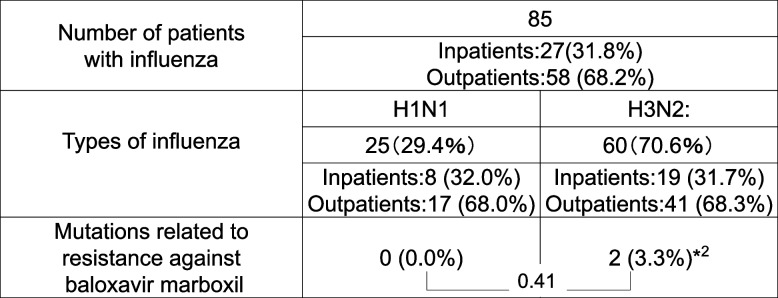
Mutations ^*1^ related to resistance against baloxavir marboxil among type A influenza isolates from patients with influenza in Kawasaki Medical School Hospital during the 2023–2024 flu season

*NS* Not Significant

^*1^The isoleucine-38 (I38) T variant of the cap-dependent endonuclease-baloxavir marboxil complex structure was detected

^*2^The two cases with the I38T variant were a 28-year-old woman and a 57-year-old man. Both were infected by their children

A total of 85 influenza type A viruses were isolated from patients at our hospital, including 27 (31.8%) in-patients and 58 (68.2%) out-patients. Of these, 25 (29.4%) were H1N1 strains, whereas the remaining 60 (70.6%) were H3N2 strains. None of the H1N1 strains harbored the I38T variant of the cap-dependent endonuclease-baloxavir complex structure, but two of the H3N2 isolates (3.3%) did. No other significant differences were observed between the influenza types. No mutant strains were found among the samples from patients with secondary influenza infections. The two patients who were infected with the I38T variant were a 28-year-old woman and a 57-year-old man. Both were infected with H3N2 influenza by their children, according to the medical histories provided by their attending physician. However, the children were not tested for mutations related to resistance against baloxavir marboxil

## Discussion

The prophylactic use of baloxavir to prevent influenza infection has been previously reported [3]. This study focused on those who had close contact with others who were infected with influenza, and we found that the percentage of patients who became infected with influenza by close contact was significantly lower among those who received prophylactic baloxavir (1.9%) compared to those who did not (13.6%). In reality, individuals may have even closer contact with others who are influenza-infected within their households than in-patients do with other infected patients in hospital settings.

As hospitalized in-patients may be at a higher risk of developing severe influenza, particularly immunocompromised individuals and pregnant women [7], our study provides valuable data regarding ways to prevent such nosocomial infections. One recent systematic review focusing on the use of antivirals as influenza therapies during the post-exposure period analyzed 33 trials of six antivirals (zanamivir, oseltamivir, laninamivir, baloxavir, amantadine, and rimantadine) [8]. All six agents achieved significant reductions in symptomatic influenza in individuals who were at high risk of developing severe disease. Among these agents, we believe that baloxavir represents the optimal therapy for this purpose. This is because baloxavir can be administered via ingestion, which has a higher compliance rate compared to inhalation.

However, caution should be exercised in cases of influenza that harbor mutations related to baloxavir resistance. One study found that the PA I38T substitution, the most common mutation related to baloxavir resistance, was prevalent among Japanese children **in**fected with H3N2 influenza viruses in 2018 [9]. This was quickly followed by a number of other similar reports over the following flu seasons [10–13] In a recent study, Chon et al. reported that two (0.8%) of the 234 total influenza A (H3N2) viruses they analyzed carried the PA/I38T substitution mutation prior to the administration of baloxavir treatment, and eight (14.8%) carried the substitution following baloxavir treatment, during the 2022–2023 influenza season in Japan [13]. All of the mutation-harboring samples in that study, other than one that was taken before the treatment, were from pediatric patients. In fact, these types of resistance-conferring substitutions have been observed much more commonly among children vs adults in several past reports [3, 14].

In our study, two of the untreated adult patients, both of whom were out-patients, were found to be infected with influenza viruses harboring the I38T mutation. However, both of these cases had most likely been infected with influenza through their children.

This study was subject to certain key limitations worth noting.

First, we did not survey the cost-effectiveness of baloxavir as a prophylactic for hospitalized in-patients. However, this aspect has been investigated in several previous reports [15–18]. Two studies investigated the economics of baloxavir vs laninamivir [15, 16], whereas another two compared baloxavir to oseltamivir [17, 18]. Baloxavir was determined to be more cost-effective than the other anti-influenza agents in all those reports. Therefore, baloxavir may be cost-effective for use as a prophylactic as well.

Our study on the efficacy of baloxavir as a prophylactic among hospitalized in-patients was a single-center observational study rather than a randomized controlled trial (RCT) or multi-center study. However, many hospitalized in-patients are considered at high risk for developing severe influenza, which may make it particularly difficult to perform an RCT on this approach in an ethical manner. Moreover, there might be few medical settings in Japan where baloxavir is being used prophylactically for in-patients because it has not been long since the drug was approved for this usage in the country.

In addition, we were unable to investigate the backgrounds of the patients we observed. As you know, patients with immunosuppression may be more susceptible to influenza infection. Therefore, detailed surveys are needed in the future.

Finally, this field of research would benefit highly from detailed investigations on influenza virus samples, including some taken from asymptomatic contacts with influenza patients, the detection of amino acid substitutions other than the I38T variant, and quantitative measurements of viral susceptibility to anti-influenza drugs such as the Effective Concentration 50 (EC_50_). In fact, few investigations have been performed in household contacts of patients with influenza patients who were asymptomatic but were confirmed to be infected through RT-PCR [3]. However, this type of testing is becoming more widespread in hospitals, as the testing of asymptomatic contacts of patients with influenza following isolation from other in-patients is considered a practical strategy to prevent secondary nosocomial infections.

Regarding amino acid substitutions conferring drug resistance in the influenza virus, almost all of the ones identified thus far have comprised the I38T variant [10, 13]. However, other mutations should be investigated in future studies, as well as the EC_50_ of baloxavir.

## Conclusions

Baloxavir was effective as a prophylactic for preventing influenza infection among the Japanese hospitalized in-patients in our study. Particular caution should nevertheless be exercised, particularly around children, in cases of influenza that harbor drug-resistance mutations, such as the I38T variant. We eagerly anticipate more data concerning the use of baloxavir as a prophylactic treatment against influenza, as well as more investigations concerning influenza mutations, in future research.

## Data Availability

All data generated or analyzed in this study are included in this published report.

## Abbreviations

RT-qPCR: Reverse-transcription-quantitative-polymerase-chain-reaction
PA: Polymerase acidic subunit
EC_50_: Effective Concentration 50
RCT: Randomized controlled trial
LNA: Locked nucleic acid
